# Comparison of Crohn’s disease-associated adherent-invasive *Escherichia coli* (AIEC) from France and Hong Kong: results from the Pacific study

**DOI:** 10.1080/19490976.2024.2431645

**Published:** 2024-11-25

**Authors:** Caroline Chevarin, Zhilu Xu, Lucas Martin, Frederic Robin, Racha Beyrouthy, Jean-Frédéric Colombel, Alexander Sulakvelidze, Siew C Ng, Richard Bonnet, Anthony Buisson, Nicolas Barnich

**Affiliations:** a Microbes, Intestin, Inflammation et Susceptibilité de l’Hôte (M2iSH), Université Clermont Auvergne/Inserm U1071, USC INRAE 1382, Clermont-Ferrand, France; b Department of Medicine and Therapeutics, Institute of Digestive Disease, State Key Laboratory of Digestive Diseases, LKS Institute of Health Science, The Chinese University of Hong Kong, Hong Kong, China; c Microbiota I-Center (MagIC), Faculty of Medicine, The Chinese University of Hong Kong, Hong Kong SAR, China; d Centre National de Référence de la Résistance aux Antibiotiques, Centre Hospitalier Universitaire, Clermont-Ferrand, France; e Department of Gastroenterology, Icahn School of Medicine, Mount Sinai, NY, USA; f Intralytix, Inc, Columbia, MD, USA; g Service d’Hépato-Gastro Entérologie, Université Clermont Auvergne, Inserm, 3iHP, CHU Clermont-Ferrand, Clermont-Ferrand, France

**Keywords:** Crohn’s disease, adherent-invasive *E. coli*, trans-ethnic study, bacterial comparison

## Abstract

Association between ileal colonization by Adherent-Invasive *Escherichia coli* (AIEC) and Crohn’s disease (CD) has been widely described in high-incidence Western countries but remains unexplored in Asian countries with a fast increase in CD incidence. In the PACIFIC study, we compared the characteristics of AIEC pathobionts retrieved from ileal biopsies of CD patients enrolled in France (FR) and Hong Kong (HK). The prevalence of AIEC was similar in France (24.5%, 25/102) and Hong Kong (30.0%, 18/60) (*p* = 0.44). No difference was observed between the two populations of AIEC regarding adhesion and invasion levels. When tested for antibiotic resistance, the proportion of AIEC strains resistant to ampicillin, piperacillin, tobramycin, and gentamicin was significantly higher in HK AIEC strains compared to French strains. AIEC strains from FR or HK population were both able to persist in the mice intestine (DSS-treated CEABAC10 mice model). Moreover, genomic analysis of 25 FR and 17 hK AIEC strains using next-generation sequencing revealed the co-existence of several virulence factors associated with enteric *E. coli* pathotypes, although no single virulence factor was significantly associated with either country of origin or AIEC status. *In vitro*, all AIEC strains (FR and HK) were sensitive to the EcoActive™ phage cocktail, suggesting that it could be a promising option to target AIEC in CD across the world.

## Introduction

Crohn’s disease (CD) is an inflammatory bowel disease (IBD) that can induce chronic inflammation of the gastrointestinal tract, leading to bowel damage and altered quality of life for patients.^1^ CD may affect different populations, including children, adults, and elderly people, regardless of geographic area or ethnicity.^2,3^ Epidemiology of CD varies according to the area of the world and can be summarized in distinguishing three distinct epidemiological stages: i) since 2020, Western countries (*i.e*., North America, Western Europe, and Oceania) display a relatively stable incidence but a sharp rise in the prevalence; ii) newly industrialized countries in Asia, Latin America, and the Middle East experience rising incidence but relatively low prevalence, and iii) in developing countries, we can observe the emergence of sporadic IBD cases.^4–6^ Epidemiological studies led in Eastern countries could be of great value to better understand the pathogenesis of CD and thus attempt to reduce its incidence. Although the exact etiology of CD remains unknown, experimental and observational data suggest that intestinal inflammation arises from an abnormal immune response to intestinal microbiota, favored by environmental factors and genetical susceptibility.^5^ Westernization of life habits could modify environmental exposure that can influence the microbiome and contribute to the genesis of CD.^6,7^ CD-associated dysbiosis is characterized by an increased number of mucosa-associated *Enterobacterales*, especially *Escherichia coli*, and a reduced overall biodiversity.^8–11^ Among these microbiota alterations, the association between ileal CD and a specific *E. coli* pathovar, adherent-invasive *E. coli* (AIEC), has now been widely demonstrated across the world.^12^ AIEC is able to adhere to and invade intestinal epithelial cells (IECs) as well as to replicate within macrophages, leading to increased levels of pro-inflammatory cytokines.^12–14^ However, owing to very different ecosystems according to the area of the world, assessing whether AIEC characteristics are similar or different across the world is paramount, while therapeutic strategies targeting AIEC (antibiotics, phages, fecal microbiota transplantation…) to treat patients with CD are currently being investigated. In this work, we aimed to compare the prevalence, the genomics, the phenotypic properties, the sensitivity to antibiotics, and bacteriophage cocktails between AIEC strains retrieved from the ileum of two different populations of patients with CD: Caucasians living in France and Chinese living in Hong Kong.

## Methods

### Ethical considerations

The study was performed in accordance with the Declaration of Helsinki, Good Clinical Practice, and applicable regulatory requirements. The French ethical committee, so-called *“Comité de Protection des Personnes (CPP) Sud-Est 6”* – France, approved the French study [AU 904]. The Chinese study was approved by the Ethics Committee of the Chinese University of Hong Kong [CRE 2014.026].

The *in vivo* experiments were carried out in strict accordance with the recommendations of the Guide for the Care and Use of Laboratory Animals of Clermont Auvergne University (Clermont-Ferrand, France). The Committee for Research and Ethical Issues of the Department of Auvergne (CEMEA Auvergne) approved the animal protocol (Permit Number: CEMEAA 2,018,031,914,539,228). Mice were housed in an animal facility of Clermont Auvergne University (Clermont-Ferrand, France) in specific pathogen-free conditions, with access to food and water *ad libitum*.

### Design of the study

Biopsies were taken from the ileum of 60 hong Kong Chinese (HK) CD patients undergoing colonoscopy at the Prince of Wales Hospital, the Chinese University of Hong Kong, and from the ileum of 102 French (FR) CD patients requiring ileo-colonoscopy at multiple IBD centers in France.^15^ Patients were included if they were 18 years or older with a diagnosis of ileal CD (ileal or ileocolonic); had active ileal disease at the time of endoscopy; and had stable CD-related medication. Patients with antibiotics, probiotics, prebiotics, or a history of enteric infection in the past 3 months were excluded. Tissues were immersed in the cell culture medium MEM supplemented with 15% sterile glycerol, snap frozen, and stored at −80°C until microbiological analysis.

### Isolation and characterization of AIEC bacteria

Ileal biopsies were crushed in phosphate-buffered saline (PBS), and the lysate was plated on Drigalski agar to isolate *Escherichia coli* colonies after 24 hours of incubation at 37°C, as previously described.^15,16^ A random selection of 45 lactose-positive colonies per sample was validated for *E. coli* species using an automated mass spectrometry microbial identification system based on Matrix Assisted Laser Desorption Ionization Time-of-Flight (MALDI-TOF) technology, according to manufacturer’s recommendations (VitekII®, Biomérieux, France). They were then cultured in 96-well microplates in a Luria-Bertani medium at 37°C, supplemented with 15% glycerol, and stored at −80°C until AIEC characterization. Each French and Chinese partner has carried out the AIEC characterization by analyzing their abilities to adhere to and invade intestine-407 epithelial cells (ATCC, CCL-6) and to survive and replicate within THP-1 macrophages (ATCC, TIB-202) by conducting antibiotic protection assays, as previously described.^14,15^ Briefly, the 45 *E. coli* strains per biopsy were pre-screened for invasive abilities by mixing equitably and extemporaneously the overnight bacterial cultures and infecting I-407 cells at a multiplicity of infection of 100 bacteria per cell. Three hours later, infected I-407 cells were exposed for 1 h to 100 µg/ml of gentamicin or 50 µg/ml of meropenem (depending on strain resistance to antibiotics) to kill extracellular bacteria. Cells were lysed using Triton 1X, and the lysate was plated on Drigalski agar to isolate surviving bacteria. AIEC phenotype of these invasive strains was validated by conducting antibiotic protection assays to confirm their abilities to adhere to and invade I-407 cells and to survive and replicate within THP-1 macrophages.

### Adhesion and invasion assays on AIEC bacteria

AIEC strains characterized from Hong Kong and from France were compared in the same experiment of adhesion and invasion to I-407 cells. As previously described,^14^ I-407 cells were seeded in 24-wells tissue culture plates at a density of 4.10^5^ cells/well and incubated for 24 h. The cells were washed twice with PBS before being infected with a multiplicity of infection of 10 bacteria per cell in 1 mL of adequate cell culture medium. After a 3-h incubation period at 37°C with 5% CO_2_, the monolayers were washed 3 times with PBS. Invasion was assessed by adding a fresh cell culture medium containing 50 µg/ml of meropenem (gentamicin was substituted due to high resistance to gentamicin of HK strains) for 1 h to kill extracellular bacteria. For both adhesion and invasion assays, the cells were lysed using Triton 1X, and the samples were diluted and plated onto Luria Bertani (LB) agar plates to determine the number of colony-forming units. All of the assays were performed 3 times in separate experiments. The results were expressed as the number in CFU/cell of adherent or invasive bacteria compared to the number of bacteria present in the initial inoculum. For all phenotypical assays, the non-AIEC *E. coli* strain, K-12, was used as the negative control, and the AIEC reference strain LF82 was used as the positive control.

### Bacterial strains and culture conditions

Four AIEC strains from each French (FR) or Hong Kong (HK) populations, selected for their belonging to B2 or D phylogroup, harboring or not S70/N78 mutations in the f*imH* adhesin gene^17^ and with the highest invasion rates in IECs among those that met the first two criteria, as well as AIEC LF82 strain,^18^ were used in the protocol of mouse infection (Supplemental Data Table S1). The strains were either naturally resistant to streptomycin or made resistant by insertion of the streptomycin-resistance encoding gene on the chromosome. Using a mobilizable mini-Tn7-based vector (pUC18R6KT-mini-Tn7T-Stp) delivered by *E. coli* MFDpir46 and *E. coli* MFDpir/pTNS3 strain encoding the *tnsABCD* genes necessary for the transposition of mini-Tn7 at the *attTn7* insertion site, the AIEC recipient strain was modified as a streptomycin-resistant mutant.^19,20^ The AIEC strains and mutants were grown in Luria Bertani (LB) broth under static and aerobic conditions overnight at 37°C. On the day of infection, bacterial cultures were harvested by centrifugation at 4 500 × g for 10 min, and pellets were resuspended in PBS at a concentration of 5 × 10^9^ bacteria/ml.

### Mouse infection protocol

CEABAC10 FVB/N transgenic male mice (6–8 weeks old) were used in an *in vivo* model mimicking colitis and Crohn’s disease.^21^ They were orally pretreated for 3 days with 2.5 g/l of streptomycin and 0.5% (wt/vol) of dextran sulfate sodium (DSS; molecular mass = 36,000 –50,000 daltons; MP Biomedicals) in drinking water to promote the accessibility of bacteria to the intestine. Twenty-four hours later, mice were challenged with 0.2 ml of 10^9^ AIEC bacteria (Figure 2a). Body weight was assessed over 10 days, and the severity of colitis was valued by the disease activity index (DAI) score ranging from 0 (healthy) to 12 (high colitis activity), as previously described.^22^ About 100 mg of fresh fecal samples were collected at different days postinfection (dpi) and homogenized in PBS, and serial dilutions were plated on LB agar medium containing 100 µg/ml streptomycin to select AIEC bacteria. After overnight incubation at 37°C, bacteria were numbered in CFU/ml. Ten days after oral infection, the mice were anesthetized with isoflurane and euthanized by cervical dislocation. Colon and ileum specimens were collected and washed. Serial dilutions in PBS were plated onto streptomycin-selective LB agar medium for overnight incubation at 37°C. AIEC bacteria associated with the tissue were counted in CFU/ml. Results reflecting the gain or loss of body weight between day 0 and day 10 are expressed as area under the body weight change curve (AUC) in percentage over 10 days. Results reflecting the score of DAI between day 0 and day 10 are expressed as area under the DAI score curve (AUC) in 10 days.

### Whole-genome sequencing

Whole-genome sequencing (WGS) was conducted at the teaching hospital of Clermont-Ferrand, France, using a next-generation sequencing platform, with the analysis criteria previously described.^23^ Briefly, DNA extraction was carried out with the DNeasy UltraClean Microbial kit (Qiagen, Hilden, Germany), and libraries were prepared subsequently using the Nextera XT Kit (Illumina, San Diego, CA, USA). The reads generated on the Illumina MiSeq system were filtered for quality using Fastp software v0.19.10,^24^ and short reads were assembled with SPAdes.^25^

### Molecular typing and virulence factor detection

*E. coli* phylogroups and multilocus sequence typing (MLST) were determined in silico according to the Clermont Typing method^26^ and Achtman’s MLST scheme.^27^ Core genome SNP-based typing (cgSNP) was performed with BactSNP v1.1.0^28^ using the *E. coli* core genome downloaded from the Enterobase website (https://enterobase.warwick.ac.uk) as a reference, as previously described.^29,30^ After filtration of recombination zones detected by Gubbins,^31^ a phylogenetic tree was inferred from the resulting alignment by maximum likelihood using RAxML.^32^ The detection of virulence factors was performed with blastx DIAMOND^33^ using a threshold of 85% amino acid identity and the *E. coli* VFDB database (http://www.mgc.ac.cn/VFs/). After alignment with Muscle,^34^ minimum spanning trees were constructed from amino acid sequences with Grapetree.^35^

### Antibiotics susceptibility testing

Identification of strains was confirmed by the Vitek MS MALDI TOF method (BioMérieux, La Balme, France). The production of extended-spectrum beta-lactamase (ESBL) was detected according to the recommendations of the Antimicrobial Committee of the French Society for Microbiology (CA- SFM, 2019 V2). Antibiotic susceptibilities were determined by the disk diffusion method according to the CA-SFM recommendations. ESBL production was confirmed by the double-disk synergy test. The following 29 antibiotics were tested: ampicillin, amoxicillin-clavulanic acid, ticarcillin, piperacillin, piperacillin-tazobactam, temocillin, cefalexin, cefuroxime, cefixime, mecillinam, temocillin, cefoxitin, ceftazidime, cefotaxime, aztreonam, cefepime, ertapenem, imipenem, amikacin, gentamicin, tobramycin, netilmicin, nalidixic acid, norfloxacin, ofloxacin, ciprofloxacin, fosfomycin, chloramphenicol, and trimethoprim (Biorad, Marnes-La-Coquette, France). These antibiotics were chosen according to the lists recommended by CA-SFM to identify most resistance mechanisms, especially those concerning beta-lactams (ESBL, carbapenemase especially).

### Phages sensitivity testing

Seven individual phages composing the EcoActive™ cocktail were isolated by Intralytix or the Institut Pasteur. They were selected for their lysing capacity against AIEC strains during the spot test assay, as described by Titecat *et al*.^36^ The seven monophages were tested against 46 AIEC strains from FR and HK at ~2 × 10^4^ and ~1 × 10^9^ PFU/mL concentrations using the spot test assay.^37^ Next, the specificity of the monophages combined to produce the EcoActive™ phage cocktail was examined against the 46 AIEC strains at the same concentrations.

### Statistical analysis

Values are expressed as percentage, mean with 95% confidence interval (CI) or as median with interquartile range (IQR). Statistical analyses were performed using GraphPad Prism 9 (version 9.3.1, GraphPad Software, San Diego, CA, USA). An unpaired Mann–Whitney test was performed for single comparisons, and the Kruskal–Wallis test with Dunn’s post hoc test was performed for multiple comparisons. A value of *p* < 0.05 was considered to be statistically significant.

## Results

### The prevalence and phenotype of AIEC associated to ileal mucosa are similar in France and Hong Kong Chinese populations

AIEC was detected in the ileal mucosa of 25 (24.5%) of 102 French patients with CD and 18 (30%) of 60 Chinese patients with CD (*p* = 0.44).

### Phenotypical properties of AIEC strains from France and Hong Kong

**Figure 1.**
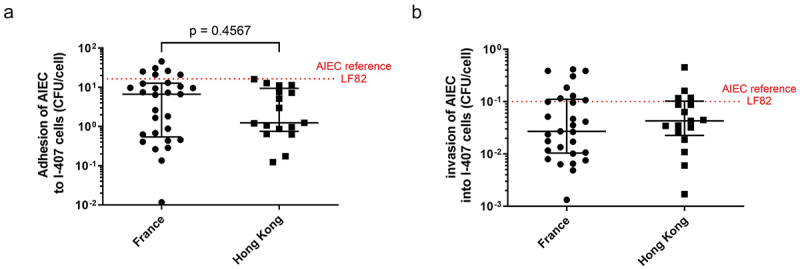
Adhesion and invasion of AIEC strains isolated from ileum of CD patients in France (*n* = 29) and Hong Kong (*n* = 17) into I-407 epithelial intestinal cell line. The number of adhered (a) or internalized (b) bacteria, to assess adhesion and invasion, respectively, was determined as described in section “materials and methods.” Results were expressed as numbers of CFU/cell and represented as median with interquartile range of triplicate experiments. Data were analyzed by Mann Whitney test, ns.

Once identified with AIEC pathovar definition using adhesion, invasion in IECs, and survival within macrophage assays, the AIEC isolates from French patients were compared with isolates from Chinese patients for their abilities to adhere and to invade intestinal epithelial cells, the main phenotypic criteria. A total of 29 AIEC isolated from 24 French patients and 17 AIEC from 16 Chinese patients were submitted to the gentamicin protection assay, modified by using meropenem due to the presence of gentamicin-resistant strains in I-407 epithelial intestinal cells. The ability of these bacteria isolated in France or in Hong Kong to adhere to IECs was not different (*p* = 0.46), with a median of 6.7 [IQR, 0.543–12.74] CFU/cell for French AIEC isolates and a median of 1.245 [IQR, 0.755–9.417] CFU/cell for Chinese AIEC isolates (Figure 1a). The median level of invasion between FR AIEC isolates (0.027 [IQR, 0.010–0.111] CFU/cell) and HK AIEC isolates (0.043 [IQR, 0.023–0.102] CFU/cell) was no different (*p* = 0.57) (Figure 1b). These results indicate that AIEC strains interact similarly with intestinal epithelial cells regardless of their geographical origin.

### AIEC bacteria from France and Hong Kong colonize the intestine of mice with similar efficacy

The DSS-treated CEABAC10 transgenic mouse model expressing *h*CEACAM6 in epithelial cells represents a preclinical model allowing to decipher the role of AIEC bacteria in inflammatory bowel diseases. This model demonstrated the ability of LF82 bacteria, the reference for AIEC strains, to adhere to the intestinal epithelium and modulate intestinal permeability.^18,21^ To investigate and compare the gut persistence of AIEC bacteria isolated from FR and HK populations, seven mice per group were challenged with one of the four different FR strains (CEA614S, CEA501S, CEA303S, and CEA615S) or one of the four different HK strains (1162d, 1186IFc, 1133a, and 1222a), and the behavior was compared to mice infected with the AIEC LF82 reference strain.

**Figure 2.**
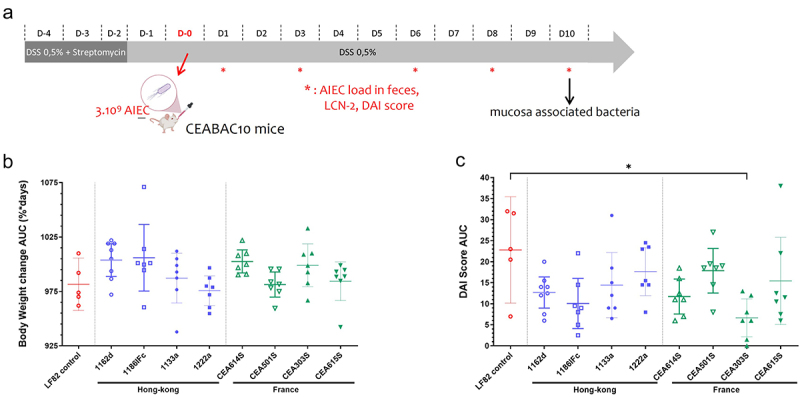
Clinical symptoms of colitis in CEABAC10 transgenic mice after an oral challenge with four FR strains (CEA614S, CEA501S, CEA303S, CEA615S), four HK strains (1162d, 1186IFc, 1133a, 1222a) and AIEC LF82 reference strain. (a) Schema of the dss-treated CEABAC10 transgenic mouse model infection protocol. AUC values represent the evolution of mice body weight (b) and DAI score (c) during 10 days after infection. *n* = 5-8 mice per group. Results are presented as mean with 95% confidence interval; Kruskal–Wallis test, *p < .05.

To analyze the consequences of AIEC colonization, body weight loss of mice orally challenged with 10^9^ bacteria was recorded. The disease activity index (DAI) score was determined at days 0, 1, 3, 6, 8, and 10 post-infection. There was a similar evolution between day 0 and day 10 in the body weight of the LF82 control group (AUC = 981.6%*days; 95% CI, 957.6–1006%*days) compared to other AIEC-infected groups (Figure 2b). No significant difference was observed in the DAI score AUC during this period of 10 days between the LF82 control group (AUC = 22.80; 95% CI, 10.15–35.45) and other AIEC-infected groups, except for the group of mice infected with CEA303S strain (AUC = 6.64; 95% CI, 2.14–11.15, *p* = .0157) (Figure 2c).

**Figure 3.**
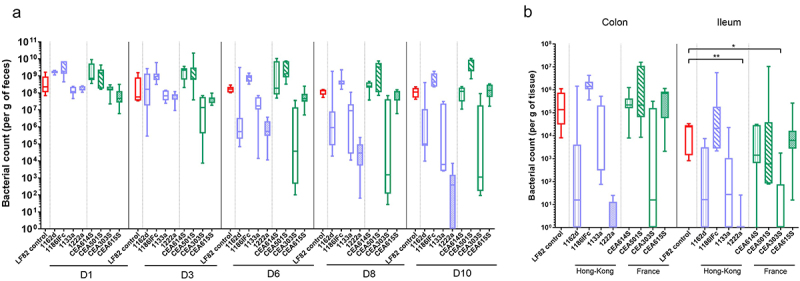
AIEC gut colonization after an oral challenge with four FR strains (CEA614S, CEA501S, CEA303S, CEA615S), four HK strains (1162d, 1186IFc, 1133a, 1222a) and AIEC LF82 reference strain in CEABAC10 transgenic mice. (a) Levels of fecal AIEC bacteria at days 1, 3, 6, 8 and 10 postinfection (Cfu/g of feces). (b) AIEC bacteria associated with the intestinal mucosa at day 10 postinfection (Cfu/g of tissue). *n* = 5-8 mice per group. Results are presented as box-plot with whiskers from minimum to maximum (red box = LF82 reference, purple boxes = hK AIEC strains, green boxes = FR AIEC strains); Kruskal-Wallis test, *p < .05, **p < .01.

The levels of fecal AIEC bacteria, reflecting the persistence of the pathobiont within the gut lumen, were assessed for 10 days and revealed that there was no difference in bacterial counts between LF82 and each of the eight FR and HK strains at days 1, 3, 6, 8, and 10 post-infection (Figure 3a). On day 10 after infection, three-fourths of the CEABAC10 mice groups that were challenged with FR strains (i.e. CEA614S, CEA501S, and CEA615S) and two-fourths of the CEABAC10 mice groups that were challenged with HK strains (i.e. 1162d and 1186IFc) still presented more than 1 × 10^5^ CFU per gram of feces, while mice challenged with LF82 strain harbored 1.1 × 10^8^ [IQR, 5.3 × 10^7^ − 2 × 10^8^] CFU/g of feces. However, 10 days after infection, 1222a strain from HK and CEA303S strain from FR showed reduced colonization at the level of 3.8 × 10^2^ [IQR, 1.0–1.5 × 10^3^] and 1.1 × 10^3^ [IQR, 1.7 × 10^2^ − 1.9 × 10^7^] CFU/g of feces, respectively.

To confirm AIEC colonization, we determined the number of mucosa-associated bacteria at euthanasia. Ten days after infection, the median colonic bacterial load of FR AIEC remained high for CEA614S, CEA501S, and CEA615S strains (respectively, 2.2 × 10^5^ [IQR, 1.6 × 10^5^ − 4.2 × 10^5^], 2.2 × 10^5^ [IQR, 6.4 × 10^4^ − 1.1 × 10^7^], and 7.0 × 10^5^ [IQR, 5.8 × 10^4^ − 8.4 × 10^5^] CFU/g of tissue) and close to median LF82 load (1.4 × 10^5^ [IQR, 6.2 × 10^4^ − 8.1 × 10^5^] CFU/g of tissue) (Figure 3b). 1186IFc HK strain was also maintained at a high level of colonization in the colon (1.4 × 10^6^ [IQR, 1.1 × 10^6^ − 2.3 × 10^6^] CFU/g). Concerning 1162d, 1133a, 1222a, and CEA303S strains at day 10, a decreased bacterial load was noticed in the colon at the median level of 1.2 × 10^2^ [IQR, 0–7.6 × 10^3^], 3.3 × 10^2^ [IQR, 3.0 × 10^2^ − 2.1 × 10^5^], 0.0 [IQR, 0–1.2 × 10^1^], and 1.5 × 10^1^ [IQR, 0–1.6 × 10^5^] CFU/g of tissue, respectively.

The median ileal bacterial load of FR AIEC remained high for CEA614S, CEA501S, and CEA615S strains (respectively, 1.4 × 10^3^ [IQR, 6.4 × 10^2^ − 2.9 × 10^4^], 5.9 × 10^2^ [IQR, 8.1 × 10^1^ − 1.0 × 10^7^], and 6.3 × 10^3^ [IQR, 2.7 × 10^3^ − 1.6 × 10^4^] CFU/g of tissue) and close to median LF82 load (2.5 × 10^4^ [IQR, 1.6 × 10^3^ − 2.9 × 10^4^] CFU/g of tissue) (Figure 3b). Similar to colonic colonization, 1186IFc strain from HK was also maintained at a high level of colonization in the ileum (2.1 × 10^4^ [IQR, 2.8 × 10^3^ − 1.8 × 10^5^] CFU/g of tissue). Consistent with colonic values, 1162d, 1133a, 1222a, and CEA303S strains displayed at day 10 in the ileum a decreased bacterial load at the median level of 1.3 × 10^2^ [IQR, 0–3.3 × 10^3^], 2.6 × 10^1^ [IQR, 0–1.1 × 10^3^], 0.0 [IQR, 0–0] and 0.0 [IQR, 0–7.5 × 10^1^] CFU/g of tissue, respectively. This suggests that most AIEC strains could colonize mice ileum to similar levels as LF82, although a heterogeneity in the levels and persistence of colonization by certain AIECs over the course of days can be observed.

### Genomic analysis revealed a majority of AIEC strains belonging to the B2 phylogroup but did not report a specific sequence typing (ST) dependent on AIEC origin

**Figure 4.**
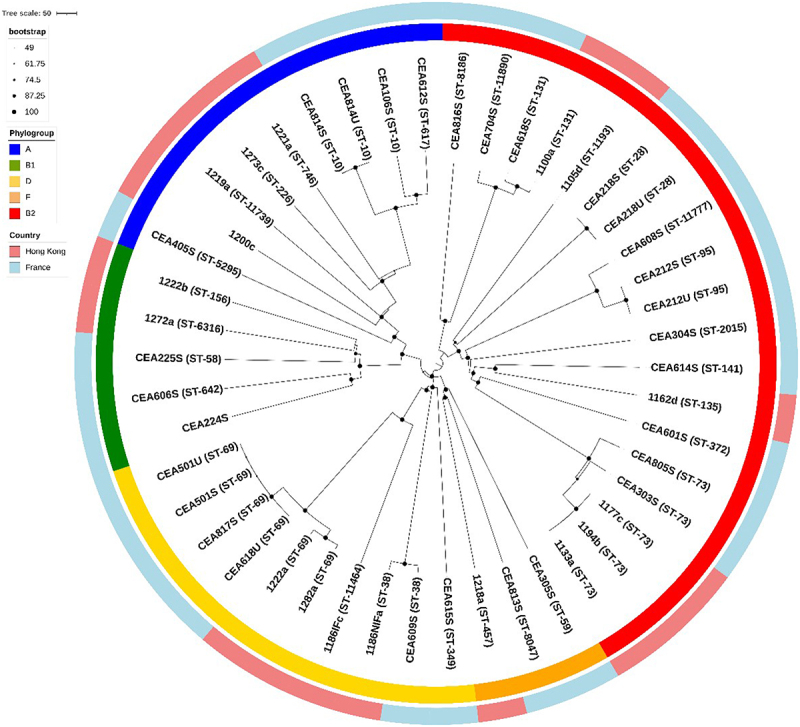
Phylogenetic analysis of AIEC isolated from French and Hong Kong Chinese CD patients. Concatenated core genome SNPs sequences were aligned and phylogenetic inferences obtained using Gubbins method and RAxML program. Sequence types (ST) correspond to Achtman’s MLST scheme. Countries where the sample collection originated are indicated by two different colors on the outer ring. All isolates were clustered into five phylogroups represented by five colors on the inner ring.

*In silico* MLST (Multi-Locus Sequence Typing) and phylogrouping were assessed from whole-genome sequencing data of 29 FR AIEC and 17 hK AIEC isolates. The core genome SNP-based phylogenetic tree representation showed that AIECs originated from the main phylogroups of *E. coli* (A, B1, B2, D, and F), belong to a wide diversity of ST lineages, and were not clustered according to their geographic origin (Figure 4). However, most AIEC strains belonged to the B2 phylogroup (19/46, 41.3%), followed by the D phylogroup (10/46, 21.7%). Within the frequent pandemic STs (for example, ST95, ST69, ST38, ST73, and ST131), AIEC samples from the two countries of origin can be found. These lineages were present among CD patients in isolated AIEC with two AIEC strains identified as ST131, two strains as ST95, five strains as ST73 belonging to B2 phylogroup, and six strains identified as ST69 and two AIEC as ST38 among D phylogroup. Of interest, the 1162d strain from the HK CD patient belonged to the same ST135 as the LF82 AIEC reference. Overall, the results show the genetic diversity within this pathovar and the absence of significant differences in the AIEC core genome from FR and HK.

**Figure 5.**
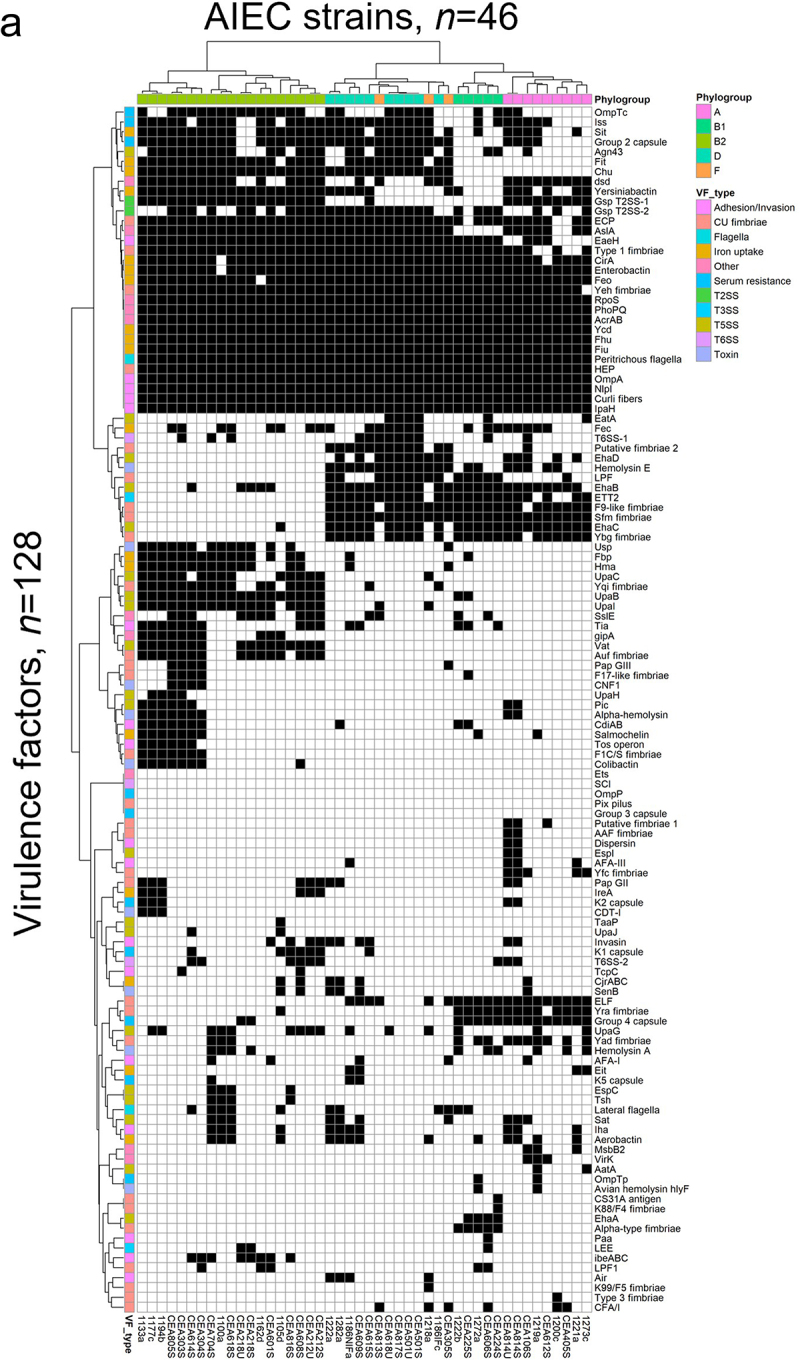
Analysis of virulence genes detected in the Illumina gene sequencing dataset of the 46 AIEC strains of this study. (a) Dendrogram analysis was performed for AIEC strains phylogenic relationships and virulence factors (VFs) relatedness using blastx DIAMOND. Black boxes indicate the presence of the gene listed on the right and white boxes, their absence. The scale on the right indicate the phylogroups and the family of the listed gene, identified by colors. (b) t-sne plot visualizing cluster assignments of AIEC strains. Strains are projected into t-sne space, with the first two t-sne components as the axes of the plot. Phylogroups denoted as distinct colors were assigned according to the Clermont method and cgSNP phylogeny.

**Figure 5.**
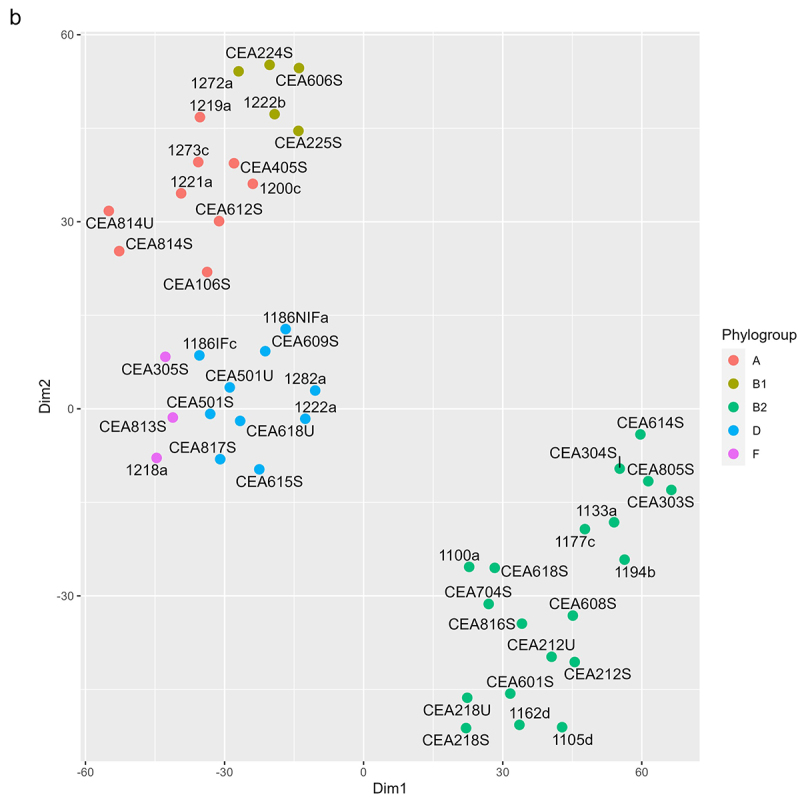
Continued.

### Genomic analysis revealed the co-existence of several virulence factors associated with enteric E. coli pathotypes across the sequenced AIEC strains

**Figure 6.**
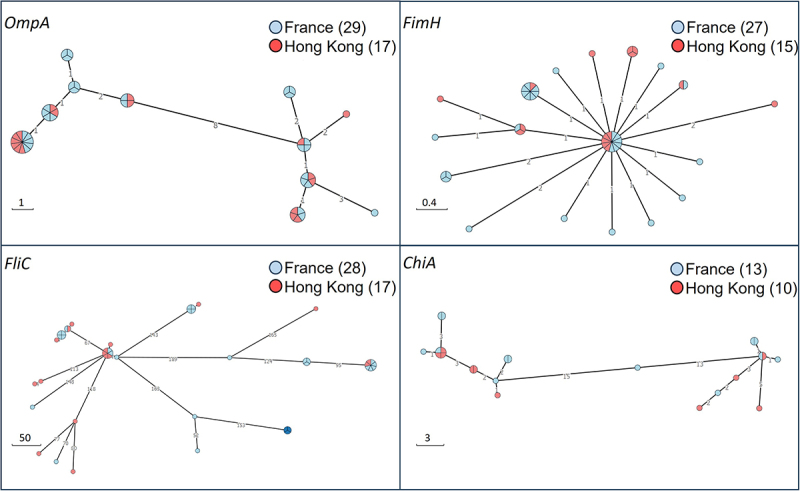
Minimum spanning trees of *ompA*, *fimH*, *fliC* and *chiA* genes variants deduced from the whole-genome sequences of the 46 AIEC strains of this study. The length of the edges is proportional to the number of amino acid substitutions (scale: substitution number), and the size of nodes to the number of strains (number of sectors). The geographic origin is indicated by the node color.

We examined the distribution of virulence factors in a gene sequencing dataset of the 46 AIEC strains of this study using a Blastx approach and the VFDB database. This procedure identified 128 virulence factors among the 46 strains (Figure 5a). Many of these virulence factors are involved in functions that are previously implicated in pathophysiology in IBD, including adhesion/invasion,^38^ flagella, iron-acquisition,^39^ serum resistance, secretion systems,^40^ and others like capsule synthesis.^41^ We observed that strains in the phylogroup B2 (44.8% from FR and 35.3% from HK collection) generally have more virulence factors than other strains and more virulence factors in common (Figure 5b). From the amino acid sequences of four virulence genes of interest (i. e. *ompA*, *fimH*, *fliC*, and *chiA*), minimum spanning trees were constructed to compare the phylogeny of the 46 AIEC strains of the study (Figure 6). We chose to focus on these specific virulence genes as they have been described as important AIEC virulence factors. In particular, previous studies showed the implication of *ompA*,^42^ *fimH*,^17^ *fliC*,^43^ and *chiA*^44^ in identifying variants in the pathogenicity of AIEC, leading to hypothesize that the presence of some AIEC-specific variants could promote the adhesion of AIEC to intestinal cells. In this study, we investigated whether AIEC isolated from HK exhibited the same variants as the strains from FR. The minimum spanning trees revealed that the majority of strains displayed the presence of *ompA*, *fimH*, *fliC* genes, and 50% of the strains, the presence of *chiA* gene. In general, the results showed the absence of one large cluster, with the AIEC isolates well-dispersed regardless of their origin. The various *fimH* variants identified were closely related as suggested by the short length of the edges (Figure 6). However, there was no observable relationship between the grouping of AIEC isolates and the country of origin.

### Antibiotics sensitivity of AIEC collection is higher in FR than in HK

The proportion of AIEC strains resistant to antibiotics was higher in HK AIEC strains compared to French strains regarding ampicillin (FR: 31%, *n* = 9; HK: 88%, *n* = 15; 2.9-fold higher, *p* = 0.0002), ticarcillin (FR: 31%, *n* = 9; HK: 88%, *n* = 15; 2.9-fold higher, *p* = 0.0002), piperacillin (FR: 31%, *n* = 9; HK: 82%, *n* = 14; 2.7-fold higher, *p* = 0.0018), tobramycin (FR: 3%, *n* = 1; HK: 70%, *n* = 12; 23-fold higher, *p* < 0.0001) and gentamicin (FR: 3%, *n* = 1; HK: 70%, *n* = 12; 23-fold higher, *p* < 0.0001) (Table 1). Over half of the total AIEC strains were resistant to ampicillin (52.3%), ticarcillin (52.3%), and piperacillin (50%) compared to sensitivity to other antibiotics tested in this study, independently of the AIEC origin. Furthermore, all isolates were susceptible to ertapenem, imipenem, amikacin, and fosfomycin. In conclusion, most FR AIEC strains were sensitive to beta-lactam antibiotics conventionally used in medical practice. In contrast, AIEC strains from HK displayed more resistance to this class of antibiotics.

**Table 1.** Proportions of AIEC strains isolated from French (*n* = 29) and Hong Kong (*n* = 17) Chinese CD patients that are resistant to specific antibiotics.

Antibiotic	France		Hong Kong		*p*-Value
	*n*	(%)	*n*	(%)	
**Ampicillin**	**9/29**	**(31)**	**15/17**	**(88,2)**	.**0002**
Amoxicillin-clavulanate	1/29	(3,4)	2/17	(11,8)	.5453
**Ticarcillin**	**9/29**	**(31)**	**15/17**	**(88,2)**	.**0002**
**Piperacillin**	**9/29**	**(31)**	**14/17**	**(82,4)**	.**0018**
Piperacillin-tazobactam	0/29	(0)	1/17	(5,9)	.3696
Mecillinam	1/29	(3,4)	1/17	(5,9)	>.9999
Temocillin	4/29	(13,8)	4/17	(23,5)	.4429
**Cefalexin**	**0/29**	**(0)**	**5/17**	**(29,4)**	.**0045**
Cefoxitin	0/29	(0)	1/17	(5,9)	.3696
**Cefixime**	**0/29**	**(0)**	**4/17**	**(23,5)**	.**0146**
**Cefuroxime**	**0/29**	**(0)**	**4/17**	**(23,5)**	.**0146**
Ceftazidime	0/29	(0)	3/17	(17,6)	.0448
Cefotaxime	0/29	(0)	3/17	(17,6)	.0448
Aztreonam	0/29	(0)	3/17	(17,6)	.0448
Cefepime	0/29	(0)	2/17	(11,8)	.1314
Ertapenem	0/29	(0)	0/17	(0)	>.9999
Imipenem	0/29	(0)	0/17	(0)	>.9999
**Tobramycin**	**1/29**	**(3,4)**	**12/17**	**(70,6)**	**<.0001**
**Gentamicin**	**1/29**	**(3,4)**	**12/17**	**(70,6)**	**<.0001**
Amikacin	0/29	(0)	0/17	(0)	>.9999
Nalidixic acid	2/29	(6,9)	5/17	(29,4)	.0832
Norfloxacin	2/29	(6,9)	3/17	(17,6)	.3429
Ofloxacin	2/29	(6,9)	5/17	(29,4)	.0832
Ciprofloxacin	2/29	(6,9)	3/17	(17,6)	.3429
Chloramphenicol	1/29	(3,4)	3/17	(17,6)	.1354
**Trimethoprim**	**4/29**	**(13,8)**	**8/17**	**(47,1)**	.**0187**
**Sulfamethoxazol-trimethoprim**	**3/29**	**(10,3)**	**8/17**	**(47,1)**	.**0100**
Fosfomycin	0/29	(0)	0/17	(0)	>.9999
**Netilmycin**	**0/29**	**(0)**	**6/17**	**(35,3)**	.**0013**

The antibiotics were chosen according to the lists recommended by the Antimicrobial Committee of the French Society for Microbiology (CA-SFM, 2019 V2).

### AIEC collections from both sides of the globe are susceptible to the EcoActive™ phage cocktail, a potential therapy

The lytic range of each of the seven phages was assessed on 29 AIEC strains obtained from FR and 17 AIEC strains obtained from HK CD patients at two concentrations: 2 × 10^4^ and 1 × 10^9^ PFU/mL. As summarized in Table 2, 25 of the 29 FR AIEC (86.2%) and 15 of the 17 hK AIEC (88.2%) were susceptible at 2 × 10^4^ PFU/mL to at least one of the monophages included in the EcoActive^TM^ cocktail. The efficacy reached 100% at the higher phage concentration [i.e., × 10^9^ PFU/mL] more commonly used in the Spot Test assay, suggesting that the component phages included in the EcoActive^TM^ phage cocktail had a strong lytic potency against AIEC strains from both FR and HK.

**Table 2.** Summary of the lytic activity of the AIEC targeting seven monophages and the EcoActive^TM^ cocktail (2 × 10^4^ and 1 × 10^9^ PFU/mL) against a panel of isolates from France (*n* = 29) and Hong Kong (*n* = 17).

	FRANCE		HONG KONG	
Phage preparation	2 × 10^4^	1 × 10^9^	2 × 10^4^	1 × 10^9^
ECML-119	28%	66%	41%	59%
ECML-1232	34%	83%	47%	76%
ECML-359	38%	72%	35%	76%
ECML-363	28%	62%	35%	76%
CLBP2	45%	76%	65%	82%
LF82P2	14%	48%	6%	71%
LF82P8	24%	66%	6%	47%
**EcoActive**	**86.2%**	**100%**	**88.2%**	**100%**

Percentages represent the number of AIECs in the total collection from each country lysed by the phage or the cocktail at each concentration.

## Discussion

In the PACIFIC study, we performed, for the first time, a direct comparison of AIEC strains retrieved from two very different geographic areas. We did not report any differences regarding adhesion and invasion properties, phylogenetic characteristics of the strains (mainly B2 phylogroup), or ability to colonize the gut in a mice model. While the antibiotics resistance profile was different between the two types of strains, we found a strong lytic potency of the EcoActive^TM^ phage cocktail against AIEC strains regardless of their origin (FR or HK), suggesting that phage therapy may offer a more promising therapeutic option to target AIEC across the world.

The PACIFIC study described for the first time the presence of AIEC among the Hong Kong Chinese population, a population of particular interest owing to a low but increasing CD incidence.^6^ The prevalence of CD patients colonized by AIEC in Hong Kong (30%)^16^ is comparable to the prevalence observed in French CD patients (24.5%).^15^ A meta-analysis from Kamali Dolatabadi *et al*.^45^ shows that the prevalence of CD patients colonized by AIEC in our study is comparable to the prevalence observed so far in different parts of the world (data published in 2004–2020). The prevalence observed in French CD patients is consistent with the prevalence of AIEC reported in CD patients from Europe and North America,^46^ suggesting that the presence of these bacteria does not depend on HK-specific factors such as different genetic backgrounds and lifestyles. Due to a significant resistance of HK AIEC strains to gentamicin, we compared *in vitro* HK and FR isolates for their invasion abilities in I-407 cells by replacing gentamicin with meropenem, a carbapenem antibiotic that does not enter eukaryotic cells^47^ in gentamicin protection assay. The absence of difference in adhesion and invasion levels between HK and FR AIEC isolates is a first indicator that AIEC properties seem comparable regardless of geographical and ethnic origin.

Thanks to our whole-genome sequencing of AIEC isolates from France and Hong Kong assessing the phylogenetic relationships between AIEC strains, we showed that major sequence types associated with AIEC phenotypes could be found in both countries. For the first time, based on the sequence typing method, we showed that AIEC strains from Asia can be related to AIEC from Europe. Moreover, a HK strain named 1162d shared the same ST as the LF82 AIEC reference isolated from French CD patients.^48^ These findings may imply that these strains have emerged from the same ancestral lineage, as is the case, for example, with diverse human Enterotoxigenic *Escherichia coli* (ETEC) isolates circulating in the human population today that have probably originated from globally widespread ETEC lineages.^49^ AIEC isolates from studies on *E. coli* isolated from patients with IBDs represent various serotypes with a large range of ST.^39,40,50,51^ Among them, the presence of pandemic STs has been reported in a few studies.^52,53^ On the other hand, numerous studies have reported the clonal dissemination of extraintestinal pathogenic *E. coli* (ExPEC) strains and five major pandemic clonal lineages of ExPEC detected in widespread infectionsin particular, urinary tract infections and bloodstream infections. These lineages include ST131, ST95, ST73, ST69, and ST38.^54–58^ In our study, these five clones were detected among AIEC strains. Martinez-Medina *et al*.^53^ reported that a subgroup of strains belonging to the AIEC pathovar is closely related to the ExPEC. The study demonstrated that 40% of the intestinal isolates belonged to ST131 and 21.7% to the ST73. Similarly, among some groups of patients with CRC, a high prevalence of AIEC ST131, ST95, and ST73 was observed.^59^ All these results suggest that some extraintestinal *E. coli* could cause intestinal inflammation or intestinal AIEC could lead to extraintestinal infections. These lineages are highly resistant to antibiotics,^55,60^ making them a major concern for public health. Focusing on phylogroups, we found that the most common phylogroup within the AIEC strains was B2. This is consistent with eight previous studies that reported the same most represented phylogroup among AIEC.^45^ In accordance with available scientific literature,^51,61^ we did not detect any virulence factor strictly associated with AIEC, but the co-existence of several virulence factors mostly associated with extraintestinal pathogenic *E. coli*. This could be explained by several hypotheses such as how AIEC emerged, the fact that the approaches used so far are not appropriate enough, or the lack of a standardized method for AIEC phenotypic characterization.^62^ Several genetic elements more frequently described in AIEC pathogenicity were also identified in our study. For example, little evidence exists on the putative role of OMPs in AIEC virulence, and Rolhion *et al*. in 2010^42^ found *ompA* amino acid variants that could be responsible for the increased invasion ability. Nonetheless, in agreement with previous study^63,64^ that found that *ompA* gene variants were similar between AIEC, IPEC, ExPEC, and non-AIEC strains, we did not detect in our study a particular variant specific to AIEC or specific to AIEC about its origin. The same report was made with *fliC*, *fimH*, and *chiA* gene variants, which are heterogeneously distributed among AIEC strains regardless of their origin.

Nonetheless, results obtained on FimH, one of the most studied virulence factors in AIEC pathotype, showed that *fimH* gene sequence variants were not so distant from each other and a group of five FR strains and six HK strains shared the same variant. The identified sequence of this variant is consensus, meaning that it is the most common and evolutionarily primary *fimH* variant. On the other hand, some strains harbored a FimH that differed from the consensus sequence by different substitutions including N70S and S78N substitutions described in the AIEC collection by Dreux and colleagues.^17^ In accordance to Camprubi-Font *et al*. work,^65^ we reinforce the idea that no particular variants were associated with AIEC origin in the present work and that no exclusive pathoadaptive changes are associated with the AIEC phenotype but could be a mark of the transition from commensalism to pathobionts in *E. coli.*

The DSS-treated CEABAC10 transgenic mouse model expressing *h*CEACAM6 in epithelial cells represents a well-known preclinical model for assessing the capacity of AIEC bacteria to adhere to the intestinal epithelium,^18^ used to study the role of environmental factors such as a Western-style diet,^66,67^ and for highlighting the efficacy of various anti-AIEC strategies, i.e., bacteriophages,^68^ anti-virulence molecules or even yeast probiotics.^69,70^ In this work, we showed that a subset of four AIEC selected per country of origin confirms the persistence of these bacteria in the mice gut, regardless of their original affiliation. This finding reinforces the fact that not only the LF82 reference strain but a majority of AIEC strains strongly interact with the intestinal mucosa.^15,71^ Nonetheless, as we observed, some strains isolated from France or Hong Kong may struggle to colonize the gut of CEABAC10 mice. Bleich *et al*.^72^ recently demonstrated that the *in vitro* AIEC pathotype definition cannot truly predict colonization phenotype *in vivo*. Indeed, it would be rather the colonization-associated features and genomic features of *E. coli* strains that convey metabolic advantages (e.g., iron acquisition and carbohydrate consumption) that lead to efficient mucosal colonization than the high level of invasion assessed *in vitro*. So, we may assume that some AIEC strains chosen in our animal experiment do not satisfy all the conditions for effective colonization without challenging the AIEC gut colonizing potential, regardless of their origin. Kittana and colleagues^73^ challenged the theory of Bleich *et al*. and showed a strong positive association between *E. coli* survival and replication in macrophages and epithelial cells *in vitro* and strain pathogenicity *in vivo*. While they observed a heterogeneity regarding the behavior of different *E. coli* strains *in vivo*, our study found the same trend regarding AIEC bacteria. It is not surprising owing to the phenotypical definition of AIEC with huge variability of genomes.

Several arguments do not support antibiotic use in the treatment of CD. First, antibiotic use has been correlated with an increased risk of CD in several epidemiological studies of high-income countries.^74^ Then, although certain antibiotics are used in the treatment of CD, their effectiveness appears to be limited to certain manifestations of the disease.^75^ They are also known to cause changes within the microbiome that can lead to mild inflammation and altered nutrient availability within the intestine.^76^ In our study, most FR AIEC strains were sensitive to beta-lactam antibiotics conventionally used in medical practice. Some works in North America and Spain reported that ileal CD-associated *E. coli* manifest resistance to commonly used antimicrobials. Dogan *et al*. published that AIEC with resistance to one or more antimicrobials were present in 6/8 (75%) AIEC-colonized ileal CD patients and that 8/13 (62%) AIEC strains from these patients were resistant to one or more of the 17 tested antimicrobials.^77^ For example, resistance to ciprofloxacin and trimethoprim/sulfamethoxazole was found in 2/6 and 4/6 AIEC-colonized ileal CD patients. Camprubí-Font *et al*. showed that the presence of *pic* virulence gene and ampicillin resistance in *E. coli* strains have a probability of 82% to be AIEC.^65^ Cho *et al*.^78^ reported that higher levels of resistance to sulfamethoxazole/trimethoprim and ampicillin were detected in CD *E. coli* strains compared to control strains (17.5% vs 4.2% and 25% vs 20.8%, respectively). They determined that 20% of CD strains were carrying resistance to 2 or more antibiotics. In contrast, their work did not detect any gentamicin resistance among the 40 tested strains, while we found a low prevalence of resistance to gentamicin among AIEC strains from FR (1/29).

In contrast, we noticed that AIEC strains from HK exhibited more resistance to this panel of antibiotics, probably due to the economic status of the country and less-controlled access to medicines than European countries. Indeed, the global human antibiotic consumption was estimated by Browne *et al*. for 204 countries from 2000 to 2018 including children with lower respiratory tract infections.^79^ They identified large variations of antibiotic consumption in high-income and upper-middle income countries with the lowest levels estimated in sub-Saharan Africa and the highest in eastern Europe and central Asia. Both inappropriate antibiotic use and lack of access to antibiotics have been reported in low- and middle-income countries as previously highlighted,^80,81^ especially in south and southeast Asia, where self-medication and non-licensed antibiotic vendors are common place.^82^ Studies conducted in China between 2000 and 2012 described a high level of outpatient-prescribed antibiotics (50.3%), and excessive prescriptions are particularly effective in lower-level hospitals and in less developed western China.^83^ Interestingly, numerous available data state the heterogeneity in consumption rates according to antibiotic classes.^79,84^ While resistance to Beta-lactam antibiotics is more commonly acknowledged,^60^ HK AIEC strains in our study were more resistant to aminoglycoside antibiotics (tobramycin and gentamicin) than FR AIEC strains. This report and the work of Dogan *et al*.^77^ suggest that antibiotics will not be effective in the global fight against AIEC. Worse, they could risk exacerbating CD since it has been demonstrated that a wide range of antibiotic classes strongly potentiated initial AIEC infection and expanded AIEC in chronically infected mice.^85^

Unlike antibiotics, bacteriophages are highly specific, infecting only a limited number of strains within a given bacterial species, and have a much smaller impact on the microbiota composition.^86,87^ In this study, we demonstrated the high potential of the EcoActive™ phage cocktail to target and lyse the AIEC strains from both FR and HK. Part of the phages in this cocktail have already proved their efficacy in decreasing the intestinal colonization of LF82 AIEC strain in wild-type and CEACAM6-expressing mice.^68^ From that point, our results strongly support using this EcoActive™ phage cocktail as a new treatment option for targeting AIEC in CD patients worldwide. However, although phage therapy has been used in Eastern Europe for several decades, it is yet to be approved as an active antibacterial treatment for human use in the European Union or the United States, given that regulatory issues and quality standards need to be appropriately addressed before approval and widespread use.^88,89^ Scientific evidence from adequately powered, double-blind, placebo-controlled, randomized clinical trials is scarce. The results of a trial enrolling patients with Crohn’s disease at Mount Sinai Hospital in New York (NCT03808103) to evaluate the use of phages targeting this bacterial subpopulation are eagerly awaited. Thus, targeting microbiota is an attractive option to treat patients with CD.

In conclusion, the PACIFIC study showed that the prevalence and phenotype of AIEC bacteria are similar in FR and HK with close phylogenetic background. Unlike antibiotics resistance profile, phage therapy seems not to be impacted by the origin of the AIEC strains and could be a more promising therapeutic option to target AIEC in CD patients across the world.

## Supplementary Material

Supplemental Material

## Data Availability

The data that support the findings of this study are available in Recherche Data Gouv at https://doi.org/10.57745/AQUNJA.
