# Supplementary material for: Comparison of Crohn’s disease-associated adherent-invasive Escherichia coli (AIEC) from France and Hong Kong: results from the Pacific study
Source: Gut Microbes. 2024 Nov 25;16(1):2431645. doi: 10.1080/19490976.2024.2431645 (PMC11601055; doi:10.1080/19490976.2024.2431645)
Supplement: Supplemental Material [file KGMI_A_2431645_SM8321.docx]

**Supplemental Data Table 1: Characteristics of the AIEC strains used *in vivo.***

| ***E. coli* Strain** | **CD patients' country of origin** | **Phylogroup** | **ST** | **S70/N78 FimH clade** | **Adhesion to I-407 cells (CFU/well)** | **Invasion into I-407 cells (CFU/well)** | **Colonic tissue-associated bacteria *in vivo* (CFU/g of tissue)** |
| --- | --- | --- | --- | --- | --- | --- | --- |
|  |  |  |  |  | **Mean (10^6^) ± SD (10^6^)** | **Mean (10^4^) ± SD (10^4^)** | **Median (10^3^) [IQR] (10^3^)** |
| CEA614s | France | B2 | 141 | + | 2.91 ± 1.73 | 15.4 ± 8.84 | 220 [160 – 420] |
| CEA501S | France | D | 69 | - | 2.93 ± 2.76 | 0.95 ± 0.38 | 220 [64 – 11000] |
| CEA303S | France | B2 | 73 | + | 0.11 ± 0.12 | 0.32 ± 0.50 | 0.015 [0 – 160] |
| CEA615S | France | D | 349 | - | 10.5 ± 14.1 | 1.65 ± 0.48 | 140 [62 – 810] |
| 1162d | Hong Kong | B2 | 135 | + | 2.91 ± 1.73 | 4.65 ± 5.84 | 0.12 [0 – 7.6] |
| 1186IFc | Hong Kong | D | 11464 | - | 1.19 ± 1.90 | 1.05 ± 1.07 | 1400 [1100 – 2300] |
| 1133a | Hong Kong | B2 | 73 | - | 4.59 ± 2.14 | 3.49 ± 2.95 | 0.33 [0.3 – 210] |
| 1222a | Hong Kong | D | 69 | - | 6.45 ± 2.68 | 1.29 ± 0.22 | 0.0 [0 – 0.012] |
| LF82 | France | B2 | ST135 | + | 6.59 ± 2.11 | 3.70 ± 0.80 | 140 [62 – 810] |

CD, Crohn’s disease; ST, sequence type; CFU, colony forming unit.
